# Dynamic regulatory mechanisms of histone methylation in plant development and environmental adaptation

**DOI:** 10.1093/hr/uhag047

**Published:** 2026-02-20

**Authors:** Sa Rina, Fan Xinyue, Sun Hongmei

**Affiliations:** Key Laboratory of Protected Horticulture of Education Ministry, College of Horticulture, Shenyang Agricultural University, Shenyang 110866, China; Key Laboratory of Protected Horticulture of Education Ministry, College of Horticulture, Shenyang Agricultural University, Shenyang 110866, China; Key Laboratory of Protected Horticulture of Education Ministry, College of Horticulture, Shenyang Agricultural University, Shenyang 110866, China; National and Local Joint Engineering Research Center of Northern Horticultural Facilities Design and Application Technology, Shenyang Agricultural University, Shenyang 110866, China

## Abstract

Histone modification is an important part of epigenetic research and plays a significant role in maintaining the stability of eukaryotic genomes, regulating gene expression, and chromatin remodeling. Histone methylation is one of the most complex modification forms in epigenetic regulation, which can occur on specific lysine or arginine residues at the tail of histones. Its biological function depends on the degree of methylation (me/me2/me3). Histone methylation involves multiple links, such as ‘writer’, ‘reader’, and ‘eraser’ enzymes, and can activate or inhibit gene transcription by recruiting various downstream effector proteins. As molecular biology techniques have advanced, significant progress has been made in fundamental research on histone methylations in plants, and researchers have gained insights into its complex multilevel regulatory mechanisms. This review systematically summarizes recent advances in the roles of histone methylation in regulating plant dormancy and germination, flowering and senescence, as well as stress responses, and proposes a cross-regulatory model integrating histone methylation with multiple signaling pathways. These insights provide a theoretical foundation for the application of epigenetic breeding strategies in horticultural crops, with the goal of enhancing stress tolerance and yield.

## Introduction

**Figure 1:**
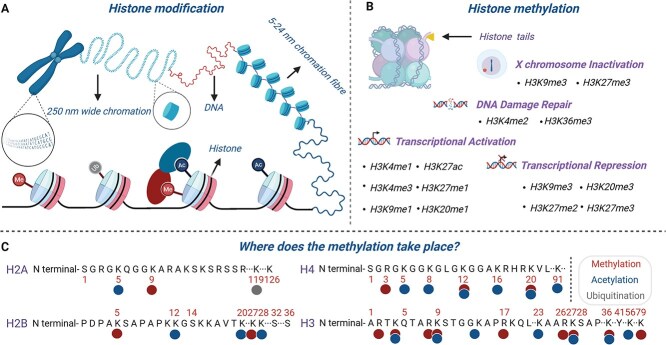
Common sites and types of histone methylation. (A) Locations and types of histone modification. (B) Common functions of different types of histone methylation modifications. (C) Common sites of different histone modifications. “The publication license for BioRender has been obtained, and the work was created by BioRender.com.”

Epigenetic regulation refers to heritable changes in gene expression status that occur without changes in the DNA sequence, and involves various molecular regulatory mechanisms, such as DNA methylation, histone modification, chromatin remodeling, and noncoding RNA [1, 2]. Within this regulatory network, post-translational modifications (PTMs) of histones include acetylation, methylation, phosphorylation, and ubiquitination. Among them (Fig. 1), the level of histone methylation is maintained by the dynamic balance of methyltransferase (HMT) and demethylase (KDM), enabling its modification state to be reversibly regulated according to cellular requirements and playing a key role in biological processes such as transcriptional regulation, heterochromatin formation, and DNA repair [3]. Histone methyltransferases (HMTs) can add methyl groups to specific lysine or arginine residues and participate in the methylation modification process in the form of ‘writers’. All known histone lysine methyltransferases contain a highly conserved SET-domain, except for the Disruptor of Telomeric Silencing 1 (DOT1)/DOT1L family members that catalyze H3K79 methylation. However, this conservation exists only for lysine methyltransferases. The typical arginine methyltransferase family PRMT belongs to the Rossmann-fold type of methyltransferase [4]. The SET domain was initially discovered in the study of the Polycomb Group (PcG) and Trithorax Group (TrxG) gene families in *Drosophila melanogaster*. Epigenetic regulation of *homeotic* (*HOM*) genes is achieved through chromatin modification and remodeling, thereby maintaining cell stability [5]. In 1997, Goodrich *et al.* [6] identified the first PcG family homologous gene, *Curly Leaf* (*CLF*), containing the SET domain in the model plant *Arabidopsis thaliana*, and found that it has a similar role in the determination of cell fate in plants and animals. Over the past 30 years, researchers have identified 43 and 22 proteins containing SET-domains in rice (*Oryza sativa*) and maize (*Zea mays*), respectively [7,8]. Zhang and Ma [9] conducted a phylogenetic analysis on 1597 SET-domain family members in the plant genome and classified them into six subfamilies: Suppressor of Variegation 3–9 (Suv); Absent, Small, or Homeotic disks 1 (Ash1); Trithorax (Trx); Enhancer of Zeste (E(z)); SET and MYND domain containing protein (SMYD), and SET domain group (SETD). Histone methylation occurs mainly at specific lysine (Lys, K) and arginine (Arg, R) residues in the N-terminal tail of histones. The functions of these modifications can be divided into inhibitory and activating markers [10]. H3K4 and H3K36 methylation is usually associated with gene activation, whereas H3K9 and H3K27 methylation is closely related to gene silencing or the maintenance of heterochromatin structure [11]. The K4, K9, K27, and K36 sites on H3 work in a coordinated or antagonistic manner to construct a complex ‘epigenetic code’ that precisely and systematically regulates multiple physiological processes in plants, from individual development to environmental adaptation.

This review not only systematically summarizes the functional characteristics of key modification sites of histone H3K4, H3K9, H3K27, and H3K36 in the regulation of plant dormancy and germination, as well as the stress response but also aims to elucidate the functional analysis of a single epigenetic marker. By integrating the research related to histone methylation with light signals, hormones and the Target of Rapamycin (TOR) pathway, a network model of histone methylation participation in multisignal cross-regulation was further constructed, and its core role as a dynamic regulatory center for integrating metabolic, environmental, and developmental signals was proposed. In addition, on the basis of the latest progress in histone methylation research in the current plant field, the feasibility of new technologies for the causal verification of chromatin function is discussed, and future research directions are proposed to provide a theoretical basis and research ideas for the precise improvement of horticultural crop traits through epigenetic markers.

## Histone methylation process

**Figure 2:**
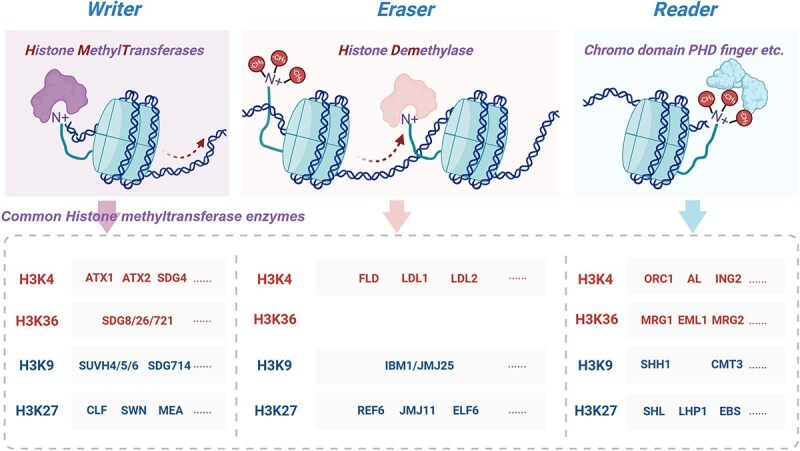
Histone methylation process. “The publication license for BioRender has been obtained, and the work was created by BioRender.com.”

The dynamic regulatory process of histone methylation involves methylation writers, readers, and erasers (Fig. 2). These enzymes affect the chromatin state and gene expression by precisely regulating the addition, recognition, and removal of methylation sites and thereby participate in various life processes in plants.

## Establishment of methylation

### H3K4 methyltransferase

The H3K4 methyltransferases containing SET domains in *A. thaliana* include mainly *A. thaliana* Trithorax (ATX1–ATX5), *A. thaliana* Trithorax-Related 3 (ATXR3), ATXR7 and the Set Domain Group 4 (SDG4), and SDG26. In *A. thaliana*, ATX1 mediates H3K4me3, promoting MRG2 recognition and binding to the *FT* promoter, and then activates *Flowering Locus T* (*FT*) expression with CO, promoting the flowering of *A. thaliana* [12]. As the core catalytic subunit of the *A. thaliana* For Complex Proteins Associated with Set1 (COMPASS) complex, ATX1 needs to combine with other conserved components (WD Repeat Domain 5, Ash2 Relative, etc.) to form a complete complex in order to exert its histone methyltransferase function [13]. Studies have shown that although the protein sequence of ATX2 is highly similar to that of ATX1, it does not have the catalytic function of H3K4me3 and only has H3K4me2 activity [14]. While ATX3/4/5 has similar domains and expression patterns and possesses both H3K4me2 and H3K4me3 methyltransferase activities [15]. ATXR3/SDG2 has been reported to play important regulatory roles in leaf development, plant height, flowering, and gametophyte development in *A. thaliana* [16,17]. ATXR7 is a Set1-like H3K4 methyltransferase in *A. thaliana*, that catalyzes the activation of the flowering repressor genes *FLC* and *Flowering Locus M* (*FLM*), *MADS Affecting Flowering 4* (*MAF4*), and *MAF5* in flowers and pollen, thereby regulating flowering time [18]. SDG4 can catalyze the methylation of H3K4 in inflorescences and pollen grains, playing a key role in pollen development. The loss of *SDG4* function will lead to a significant reduction in the methylation level of H3K4 in the pollen nutrient nucleus, thereby inhibiting the growth of pollen tubes [19]. The loss of *SDG26* function significantly reduces the H3K4me3 and H3K36me3 levels in the promoter region of the *Suppressor of Overexpression of Constans 1* (*SOC1*), resulting in a late-flowering phenotype in *A. thaliana* [20].

### H3K36 methyltransferase

SDG8 and SDG26 usually mediate varying degrees of methylation modifications at the lysine 36 position of histone H3. In research on *A. thaliana* flowering, SDG8 is a key enzyme that catalyzes H3K36me2/me3. In the *sdg8* mutant, the levels of H3K36me2/me3 on the chromatin of target genes such as *FLC*, *MAF4*, and *MAF5* significantly decreased, whereas H3K36me1 significantly increased, resulting in a decrease in the expression of these flowering inhibitory genes, and the plants exhibited early-flowering phenotype. In contrast, the absence of *SDG26* does not affect the methylation level of H3K36, but the loss of its function relieves the inhibitory regulation of target genes, leading to an increase in the expression of key flowering inhibitory genes such as *FLC*, *MAF4*, and *MAF5*, thereby causing the plant to exhibit a late-flowering phenotype [21]. In *O. sativa* L., SDG721 regulates the expression of the salt stress response gene *High-affinity* K^+^  *Transporter 1;5* (*OsHKT1;5)* by catalysing H3K36me3, thereby maintaining intracellular ion homeostasis and enhancing plant salt tolerance [22].

### H3K9 methyltransferase

H3K9, a key marker of heterochromatin formation, is catalyzed mainly by methyltransferases such as SDG714, Suppressor Of Variegation 3–9 Homolog 5(SUVH5), SUVH6, and SUVH4/Kryptonite (KYP). The SU(VAR)3–9 protein in *D. melanogaster* is the first H3K9-specific histone methyltransferase to be discovered [23]. Kryptonite (KYP), a homolog of SU(VAR)3–9 (SUVH4), was the first H3K9 methyltransferase identified in the model plant *A. thaliana* [24]. KYP/SUVH4 not only catalyzes monomethylation at H3K9 sites but also catalyzes dimethylation, but also lacks trimethylation activity [25]. The gene sequences of SDG714 and KYP/SUVH4 are highly similar, and they share similar methyltransferase activities and specificities [26]. In addition to KYP/SUVH4, SUVH5/6 in the same family has been demonstrated to have H3K9 site methylation activity *in vitro*; however, a certain degree of functional redundancy is noted among the three [27].

### H3K27 methyltransferase

Polycomb repressive complex 2 (PRC2) is a widely present epigenetic regulatory complex in plants that plays a role in transcriptional repression during plant growth, development, and environmental responses [28]. The targeting mechanism of PRC2 can be divided into two modes: those dependent on transcription factors and those independent of transcription factors. In *A. thaliana*, 12 proteins homologous to the core subunits of *D. melanogaster* PRC2 have been identified. Among them, the E(Z) family homologous proteins CLF, Medea (MEA), and Swinger (SWN) can interact with chromatin remodeling factors and are localized to target sites independently of transcription factors [28, 29, 30]. The Su(z)12 homologous proteins Embryonic Flower (EMF2), Fertilization Independent Seed 2 (FIS2), and Vernalization 2 (VRN2) need to indirectly bind to chromatin through transcription factors [31,32,33]. Additionally, the Esc homologous protein fertilization-independent endosperm (FIE) and five p55 homologous proteins, MSI1/2/3/4/5, are important PRC2 core subunits [34,35]. Overexpression of the PRC2 component *Multicopy Suppressor of Ira 1* (*SIMSI1*) in tomato (*Solanum lycopersicum* L.) leads to an increase in H3K27me3 levels and significantly delays the fruit ripening process [36]. In addition to PRC2, other H3K27 methylation regulatory mechanisms have been documented in plants, such as H3K27 monomethylation mediated by ATXR5/6 (H3K27me1), whose loss of function results in disordered heterochromatin structure and gene silencing relief [37].

## Specific recognition of methylation

Histone methylation reader enzymes are the core executor in the signal transduction of histone methylation. It can recognize specific methylated marks on histones through specific protein domains (such as the PHD finger domain, the chromodomain, the SAWADEE domain, and the Agenet domain) and further recruit chromatin remodeling complexes, transcriptional regulatory factors, etc., converting the epigenetic marks into executable biological functions, and achieving spatiotemporal specific regulation of gene expression [38]. The PHD finger domain is widely involved in the growth and development process of plants as well as the response to stress. It recognizes and binds to the epigenetic modification of the histone H3 tail and functions as a histone reader. Studies have shown that all members of the Alfin1-like (AL) family in *A. thaliana* that contain the PHD domain can recognize H3K4me3 and participate in the regulation of gene expression [39]. Furthermore, the ING family member ING2 can also recognize H3K4me3 through its PHD domain, directing the histone acetyltransferase (HAT) and histone deacetylase (HDAC) complexes onto chromatin and thereby regulating chromatin remodeling and gene expression [40, 41]. ORC1 can also activate the transcription of target genes through its domain containing a PHD finger [42]. Deletion or mutation of the PHD domain in the *Atric H Interaction Domain 5* (*ARID5*) gene leads to flowering and developmental defects in plants. Compared with wild type plant, *arid5* mutant plants are smaller and flower earlier [43]. MRG1 and MRG2 regulate the flowering time of *A. thaliana* by recognizing H3K4me3 and H3K36me3 through the chromodomain [12]. CMT3 containing a chromodomain can ensure its precise localization on the nucleosome of H3K9me2, thereby initiating DNA methylation [44]. Heterochrome protein 1 (LHP1) is the core effector factor in the epigenetic silencing pathway mediated by Polycomb inhibitory complex 2 (PRC2) and is responsible for maintaining the gene silencing state by H3K27me3. The like heterochromatin protein 1 (LHP1) protein of *A. thaliana* recognizes the H3K27me3 site of the target gene *FLC* through the chromodomain, mutation of the chromodomain weakened the ability of LHP1 to bind to *FLC* rich H3K27me3 [45]. The SlLHP1b protein in tomatoes recognizes *ACC Synthase 2*/*4* (*ACS2*/*4*), *ACC Oxidase 1* (*ACO1*), and *Phytoene Synthase 1* (*PSY1*) with inhibitory markers of H3K27me3. On the target genes of *Pectate Lyase* (*PL*), *Ripening Inhibitor* (*RIN*), and *Non*-*Ripening* (*NOR*), thereby inhibit the expression of these genes and ultimately delay fruit ripening [46]. In addition to the reader enzymes containing the classical domains, the plant-specific reader enzymes short life (SHL) and early bolting in short days (EBS) can also specifically recognize the H3K27me3 mark through their bromo adjacent homology (BAH) domains and further recruit the EMF1 protein to form a BAH–EMF1 complex [47]. In *A. thaliana*, BAH domain-containing transcriptional regulator 1 (BDT1), EBS, and SHL are highly homologous and can specifically recognize H3K27me3 to regulate the flowering time of plants [48]. Sawadee homeodomain homolog (SHH1) recognizes the H3K9 methylation status (me1/2/3) depending on the presence of H3K4 or H3K4me1. When the degree of H3K4 methylation increases (H3K4me2/3), its binding affinity with H3K9me2 is significantly reduced [49]. In *A. thaliana*, early meiocyte like 1 (EML1) recognizes and binds to H3K36me3, through its Agenet domain, thereby regulating chromatin structure and gene expression [50].

### Removal of methylations

The main eraser enzyme is histone demethylase (KDM), which removes the methyl modification of histones through peroxidation or demethylation to restore chromatin plasticity. There are two main types of demethylases (KDMs): (i) The KDM enzyme family contains the Jumonji C (JmjC) domain. There are 21 and 20 proteins containing the JmjC domain in *A. thaliana* and *O. sativa* L., respectively, and these proteins are divided into five subfamilies (KDM5/JARID1, KDM3/JHDM2, KDM4/JHDM3, JMJD6, and JmjC domain-only groups). This family has extensive substrate specificity and biological functions [42]. Increase in BONSAI Methylation 1(IBM1/JMJ25) is a specific demethylase of H3K9me1/2, and its mutation results in an abnormal increase in H3K9me2 and DNA methylation [51,52]. In *A. thaliana*, Relative of Early Flowering 6 (REF6), Early Flowering 6 (ELF6), and JMJ11/12/13/30/32 are the main demethylases of lysine 27 at histone H3 [53,54]. Among them, REF6/JMJ12 has the ability to remove H3K9me3 in addition to erasing the H3K27me3 mark [53]. Taken together, these results reveal the diversity and importance of the JmjC domain protein family in epigenetic regulation [55]. (ii) The KDM1 enzyme family (LSD1/LSD2) does not depend on the JmjC domain. In *A. thaliana*, Flowering Locus D (FLD) and LSD1-Like 1/2/3 (LDL1/2/3) belong to the homologous protein family of human lysine-specific demethylases (LSD1) [56, 57]. Among them, the amino acid sequence similarity between FLD and LDL1 and LDL2 is >65%, the similarity between LDL3 and other members is relatively low [58]. Functional studies have shown that *LDL1/2* and *FLD* inhibit *FLC* expression by reducing the H3K4 methylation level in its promoter region. Further mechanistic studies have shown that LDL1 specifically removes the monomethylation and dimethylations (H3K4me1/2) of lysine 4 in histone H3. This property is highly conserved in the catalytic properties of its human homologous protein LSD1 [59].

## Histone methylation is involved in plant growth and development

### Dormancy and germination

**Figure 3:**
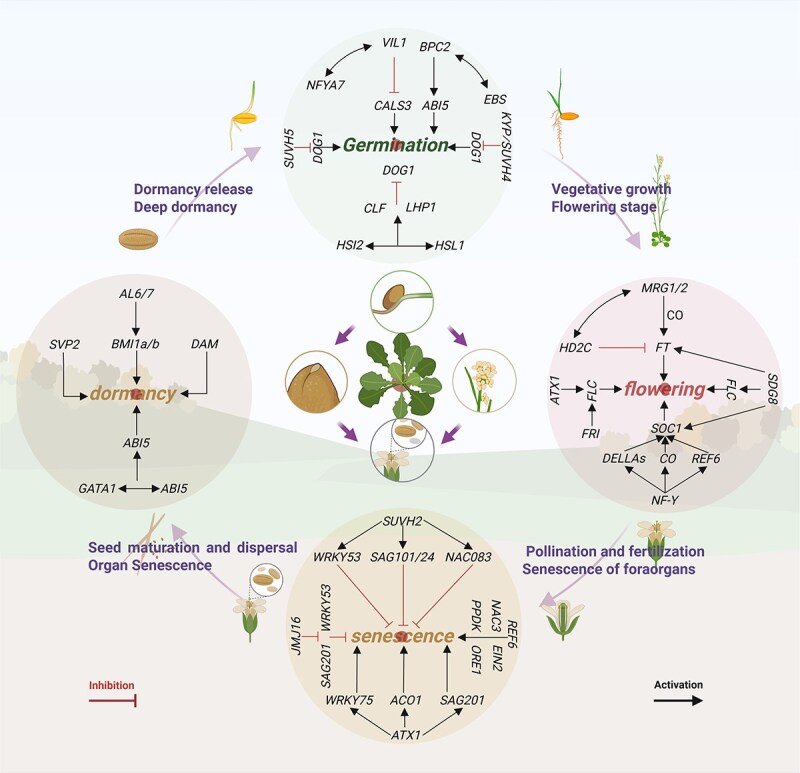
Histone methylation is involved in plant growth and development. “The publication license for BioRender has been obtained, and the work was created by BioRender.com.”

Dormancy and germination, as key physiological transformation processes in the plant life cycle, are jointly and finely regulated by external environmental factors, endogenous hormones, transcription factors, and epigenetic modifications (Fig. 3). Some epigenetic modification complexes can be recruited by specific transcription factors and thereby change the chromatin structure. Moreover, the epigenetic modification state can also regulate the binding ability of transcription factors to downstream target genes through a complex regulatory mechanism during the maintenance and release of seed dormancy. In the *TaGATA1* overexpression line of wheat (*Triticum aestivum* L.), H3K27me3 levels on the *TaABI5* promoter decreased significantly. Conversely, transient overexpression of *TaELF6*-*A1* reduced the methylation level of the *TaABI5* promoter, thereby increasing the expression of *TaABI5* and leading to increased seed dormancy [60]. *Delay of Germination 1* (*DOG1*), which was originally discovered in the model plant *A. thaliana*, has been widely identified as a switch gene for seed dormancy, and its expression level directly affects seed germination [61]. In *A. thaliana*, *KYP/SUVH4* inhibits the transcription of *DOG1* in seeds and promotes the transformation of seeds to the germinating growth state, whereas *kyp*/*suvh4* mutant seeds exhibit a significantly increased dormancy phenomenon. This inhibitory function is functionally redundant among its family members (SUVH5), but SUVH5 does not act in the same direct modification mode, but is indirectly regulated by recognition of DNA methylation [62, 63]. Similarly, the *hsi2*-*hsl1* double mutant presented a significant increase in the transcription level of *DOG1*, accompanied by increased seed *A. thaliana* dormancy. Further studies have shown that High-Level Expression of Sugar Inducible Gene 2 (HSI2) and High-Level Expression of Sugar Inducible Gene 2-LIKE 1 (HSL1) can form protein dimers. CLF and LHP1 are recruited, and H3K27me3 markers are subsequently deposited, resulting in the downregulation of *DOG1* expression [64]. The basic penta cysteine (BPC) transcription factor has been confirmed to recruit PRC2 to regulatory regions of genes involved in development and cell proliferation and other related genes by identifying GAGA *cis* elements and mediating H3K27me3 deposition to achieve gene silencing [65]. During the dormancy period of *Gladiolus hybridus* bulbs, GhBPC2 recruits PRC2 components such as Fertilization Independent Endosperm 2 (GhFIE2)、GhMSI1 and the chromatin reconstitution factor GhEBS to mediate the expression of *ABA-Insensitive 5* (*GhABI5*), thereby regulating the release of bulb dormancy [66]. However, not all PRC2 members catalyze histone methylation. Noncatalytic subunits such as Vernalization 2/5 (VRN2/5), in addition to the core catalytic subunit, are crucial for maintaining the stability and function of the PRC2 complex. Pan *et al.* [67] studies have found that Vernalization-Insensitive 3-LIKE 1 (VIL1) in *Lilium oriental* ‘Siberia’ is not only a homologous protein of *A. thaliana* VIN3, but also an important component of the PRC2 complex. Nuclear Transcription Factor Y Subunit A 7 (LoNFYA7) can recruit LoVIL1 and form a complex with it, increasing the H3K27me3 level at the *Callose Synthase 3* (*LoCALS3)* target site of the key gene encoding the enzyme for downstream callose synthesis. Therefore, the expression of *LoCALS3* is inhibited, and the growth and transformation of the terminal buds of lily bulbs are promoted. In addition to exploring the effects of H3K27me3 mediated by the PRC2 complex on plant dormancy, researchers have reported that the Alfin-like 6/7 (AL6/7) protein in *A. thaliana* functions by recognizing H3K4me3. AtBMI1a/b is recruited to transform the active transcriptional chromatin state of seed development-related genes into an inactive state, which mainly depends on the PHD domain [68]. In the early stage of bud dormancy in kiwifruit (*Actinidia chinensis*), H3K4me3 is significantly enriched in the translation initiation region (ST) of *Short Vegetative Phase 2* (*SVP2*), resulting in the early release of bud dormancy [69]. This expression pattern is similar to the regulatory mechanism of the *Dormancy Associated MADS-box* (*DAM*) observed in *Prunus persica* and *Pyrus pyrifolia* trees. These findings suggest that H3K4me3 is also functionally conserved in the dormancy regulation of perennial plants [70,71].

## Flowering and senescence

Flowering and senescence are two crucial stages in the life cycle of plants. These stages not only serve as turning points in growth and development but also play vital roles in ecological adaptation and species continuation. In recent years, with the development of molecular biology techniques, the mechanism by which histone methylation regulates the flowering and senescence processes of plants has gradually been elucidated (Fig. 3). SDG8 maintains high expression of the flowering repressor gene *FLC* by catalysing H3K36me2, thereby delaying flowering. In *sdg8* mutants, *FLC* expression is significantly reduced, leading to the activation of downstream flowering-promoting genes, including *FT* and *SOC1*, and resulting in an early-flowering phenotype [72]. BDT1 recognizes H3K27me3 in the promoter regions of key flowering genes *FT*, *SOC1*, *Agamic*-*like 6* (*AGL6*), and *Fruitfull* (*FUL*). Further recruitment of PHD1-6 and C-terminal domain phosphatase-Like 2 (CPL2) inhibits the expression of key flowering genes, thereby preventing early flowering of *A. thaliana* [48]. The daylight length (photoperiod) plays a decisive role in regulating the transition of plants from vegetative growth to reproductive growth [73]. In *A. thaliana*, the photoperiodic pathway is regulated by the transcription factor CONSTANS (CO) and by Flowering Locus T (FT). CO can positively regulate the expression of flowering related genes such as *FT* and *SOC1*. This activation of downstream flowering genes under long-day conditions is a key factor that promotes plant flowering [74]. On this basis, Bu *et al.* [12] further explored the CO-mediated long-day flowering mechanism and reported that MRG1 and MRG2 reader enzymes in *A. thaliana* could be recruited by CO proteins to the *FT* promoter region, thereby activating *FT* expression by specifically recognizing H3K4me3 and H3K36me3 in this region. In the *mrg1*/*mrg2* double mutant, the expression level of *FT* was significantly downregulated, and the *A. thaliana* presented a late-flowering phenotype under long-day conditions. In addition to promoting gene activation, MRG1/2 can provide a platform for chromatin to recruit the histone deacetylase 2C (HD2C) to inhibit the transcription of *FT*. This mechanism enables the activity of the photoperiodic flowering pathway to be regulated precisely throughout the circadian cycle, ensuring that the flowering signal is coordinated with the circadian rhythm [73]. In addition to the photoperiodic pathway, plants need to integrate multiple environmental and internal signals to accurately regulate flowering time. Studies have shown that the NF-Y transcription factor complex is key in mediating the interactions between these signals and epigenetic markers [75]. The NF-Y complex can recruit REF6 to remove the H3K27me3 inhibitory marker on the *SOC1* promoter and promote the expression of *SOC1* [76]. Plants can activate the TOR kinase through the accumulation of glucose. Activated TOR further phosphorylate the FIE protein and increase PRC2 activity, thereby increasing H3K27me3 level at the *FLC*, inhibiting FLC expression and promoting *A. thaliana* flowering [77]. This mechanism can also directly relieve the inhibition of the flowering integration gene *FT*, ultimately promoting plant flowering [28]. CLF, SWN, VRN2, FIE, and MSI1 are core members of the PRC2 complex. CLF regulates the flowering time by catalysing H3K27me3. Under short-day conditions, the *clf*-*2* mutant of *A. thaliana* shows a dwarfing phenotype compared with the wild type and has an earlier flowering time [6]. *VRN2* can stably maintain the inhibitory state of *FLC* after cold treatment. Although the expression of *FLC* in *vrn2* mutants was temporarily downregulated after 4°C low-temperature vernalization treatment, the *FLC* level rose again after returning to normal temperature [31]. Further research revealed that the PRC2 complex combines with VIN3 to form the PHD–PRC2 complex, increasing the level of H3K27me3 in the chromatin of *FLC* and resulting in gene silencing [78]. When the low-temperature period ends, *VIN3* degrades rapidly, and the VRN complex works in synergy with the H3K27me3 readers EBS, SHL, and LHP1 to maintain the silenced state of *FLC* [79]. The Frigida (FRI) protein complex activates the expression of *FLC* and maintains its high level of accumulation before vernalization, ensuring that *A. thaliana* does not flower prematurely before experiencing low temperatures [80]. ATX1 in *A. thaliana* is a known writer of H3K4me3, it activates *FLC* expression by depositing H3K4me3, thereby regulating flowering time. In the *atx1* mutant, the transcription level of *FLC* was significantly decreased, resulting in an early-flowering phenotype [14]. In carnation (*Dianthus caryophyllu*s L.), DcATX1 promotes the expression of *WRKY DNA Binding Protein 75* (*DcWRKY75*), *1*-*Aminocyclopropane*-*1*-*Carboxylate Oxidase 1* (*DcACO1*) and *Senescence-Associated Gene 12* (*DcSAG12*) by catalysing H3K4me3, thereby accelerating the process of petal senescence. Knockout of *DcATX1* can significantly delay ethylene-induced petal senescence [81]. In *A. thaliana*, *SUVH2* overexpression increased the H3K27me2/3 levels of *WRKY53*, *SAG101*/*12*/24, and *N*-*Acetylcysteine Aminohydrolase-Like 08* (*NAC083*), ultimately delaying the senescence process of leaves. These findings indicate that histone methylation also plays an indispensable role in leaf senescence [82]. Using ChIP-seq technology, Brusslan *et al.* [83] compared the histone modification levels in the leaves of *A. thaliana* on the 23rd and 52nd days after sowing and reported that significant H3K4me3 enrichment markers were not detected in ~50% of Senescence Up-Regulated Genes (SURGs). In subsequent studies, on the 29th day after sowing (when senescence began), low levels of H3K4me3 and H3K9ac premarkers were already present near the transcription initiation sites of 78% of SURGs [84]. These findings reveal the dynamic changes in histone modification during leaf development and senescence and its potential regulatory effect on gene expression. JMJ16 delays the senescence process of *A. thaliana* leaves by dynamically regulating the H3K4me3 levels on target genes *WRKY53* and *SAG201* and inhibiting their premature expression in the early stage of leaf development [85]. This mechanism reveals the key role of demethylase JMJ16 in the regulation of leaf senescence. The demethylase REF6 directly upregulates the target genes *Ethylene insensitive 2* (*EIN2*), *Oresara 1* (*ORE1*), and *Pyruvate Phosphate Dikinase* (*PPDK*), *Phytoalexin Deficient 4* (*PAD4*), and NAC3 promote the senescence of *A. thaliana* leaves. Loss of REF6 function increased H3K27me3 levels in all Senescence-Associated Genes (SAGs) [86].

## Drought stress

**Figure 4:**
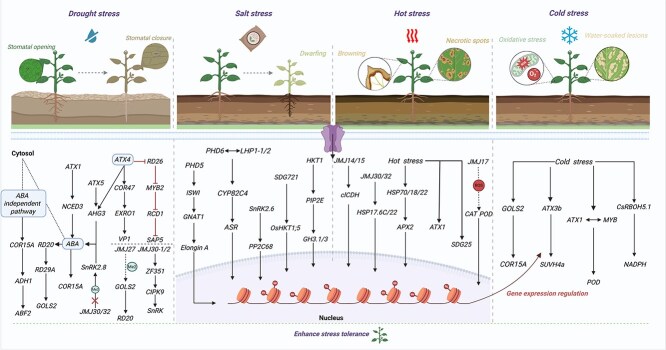
Histone methylation regulates plant environmental responses. “The publication license for BioRender has been obtained, and the work was created by BioRender.com.”

Plants adapt to environmental changes through a series of complex physiological and molecular mechanisms (Fig. 4). The establishment and maintenance of H3K4me3, an active transcriptional marker, are crucial for the continuous activation of drought response genes. Under drought stress, H3K4me3 in the promoter regions of *Response to Dehydration 29A* (*RD20*/*29A*) and *Galactinol Synthase 2* (*AtGOLS2*) in *A. thaliana* was significantly enriched. When the stress ended, H3K4me3 enrichment gradually decreased [87]. *ATX1* activates the expression of the *Nine*-*Cis*-*Epoxycarotenoid Dioxygenase 3* (*NCED3*) and *Cold-Regulated 15A* (*COR15A*) genes by increasing the level of H3K4me3. This activates the abscisic acid (ABA) signaling pathway, enhancing the drought resistance of *A. thaliana*. In the *atx1* mutant, the deposition levels of H3K4me3 on the ABA-independent genes *Alcohol Dehydrogenase 1*(*ADH1*), *ABRE Binding Factor 2* (*ABF2*), and *COR15A* were significantly lower than those in the wild type [88]. Aba-hypersensitive Germination 3 (AHG3) is an essential negative regulatory factor in the process of ABA signal transduction. ATX4 and ATX5 positively regulate *AHG3* expression by maintaining H3K4me3 levels in the *AHG3* promoter region. *AHG3* transcriptional levels decreased in *atx4* and *atx5* mutants, resulting in ABA hypersensitivity and drought-tolerant phenotypes in *A. thaliana* mutants [89]. TaATX4 mediates H3K4me2 and H3K4me3, negatively regulating the ABA signaling pathway in wheat (*T. aestivum* L.). Transcriptome sequencing, revealed that *TaCOR47*, *Expansin Related Protein* 1 (*TaEXRO1*), *Viral Protein 1* (*TaVP1*), and *Hordeum Vulgare ABA*-*induced 22*-*like protein D* (*TaHVA22D*) were upregulated in the *atx4* mutant; moreover the expressions of *TaRD26*, *Myeloblastosis 2* (*TaMYB2*), *Radical Induced Cell Death 1* (*TaRCD1*), and *Stress-Associated Protein 5* (*TaSAP5*) was downregulated. It was further verified that *TaATX4* regulated the drought resistance of wheat by regulating the expression of ABA and drought stress response genes [90]. This process of establishing and maintaining the continuous activation of drought response genes can also be catalyzed by conserved Compass-like histone methyltransferase complexes. In poplar (*Populus trichocarpa*), researchers identified a PtrSDG2-1-COMPASS methyltransferase complex that interacts with PtrAREB1–2 to form a protein tetramer. This complex activates the transcription of downstream genes, including *PtrHox2*, *PtrHox46*, and *PtrHox52*, by catalysing H3K4me3, thereby significantly enhancing poplar tolerance to long-term drought stress [ 91]. Demethylase JMJ27 positively regulates the drought stress response of *A. thaliana* by reducing the level of H3K9me2 to avoid silencing of the genes *GOLS2* and *RD20* [92]. This demethylation counteracted the transposon silencing mediated by heterochromatin, providing a more complete mechanism background for understanding the chromatin remodeling effect of JMJ27 in response to stress. Wang *et al.* [93] identified a demethylase named *Drought Tolerance* (*DT2*) that contains a JmjC domain in *O. sativa* L., and can specifically remove H3K9me2. In the *dt2* mutant, the methylation level at the *ZRT*/*IRT*-*like Protein 26* (*OsZIP26*) site increased, the expression of *OsZIP26* was inhibited, which further led to a decrease in the expression of *Basic Helix*–*Loop*–*Helix 048* (*bHLH048*), an increase in *NCED2* expression, an increase in endogenous ABA content, and increased drought tolerance. JMJ30/32 activate the expression of *Snf1-Related Protein Kinase 2.8* (*SnRK2.8)* by removing H3K27me3, thereby enhancing the ABA-dependent drought response [94]. Wei *et al.* [95] was the first to reveal that the *Zinc Finger Protein 351* (*GmZF351*) gene in *Glycine max* L. directly binds and regulates the expression of downstream *Calcineurin B-like protein-interacting protein kinase 9* (*GmCIPK9*) and *GmSnRK*, promoting stomatal closure to enhance drought resistance. Among them, the specific activation of *GmZF351* depends on the H3K27me3 mediated by GmJMJ30–1/2. In *O. sativa*, JMJ710 can remove H3K36me2 and inhibit the expression of the *MYB48*–*1*. Overexpression of *JMJ710* leads to drought-sensitive phenotypes, while RNAi and CRISPR knockout mutant lines all exhibit drought tolerance [96]. In apple (*Malus domestica*), the *RADiation-sensitive 5B* (*MdRAD5B*) gene promotes the expression of *MdSnRK2*.*6* through chromatin remodeling function, and by interacting with the recognition protein MdLHP1 of H3K27me3, it downregulates the expression of the drought-responsive gene *UDP-glycosyltransferase 71B6* (*MdUGT71B6*), thereby enhancing drought tolerance [97].

## Salt stress

Soil salinization, which results in a high concentration of sodium ions (Na^+^) and high pH, causes complex stress effects on plant growth and development (Fig. 4). Histone modification optimizes the adaptation mechanism under salt stress by regulating the transcriptional response of plants, and simultaneously promotes the expression of genes related to osmotic stress and ion homeostasis [98]. After 6 days of pretreatment with 0.3% NaCl, soybean (*G. max* L.) seedlings exhibited a significant downregulation of the ABA signaling receptor gene *Pyrabactin Resistance 1*-*Like 4* (*PYL4*) following subsequent exposure to high salinity stress (0.9% NaCl) for 1 h, whereas the transcript levels of *Protein Phosphatase 2C68* (*PP2C68*) and *SnRK2*.*6* were significantly upregulated under high-salt stress. This pattern of expression changes was closely associated with alterations in H3K4me3 levels [99]. It has been reported that *G. max* L. contains a total of six Alfin1-like proteins [100], among which GmPHD1-GmPHD5 all have transcriptional inhibitory properties [101]. GmPHD5 physically interacts with the GCN5-related N-acetyltransferase family protein 5 (GCN5), GmElonginA, and Imitation SWI/SNF (GmISWI) and jointly participates in transcriptional regulation process of *G. max* L. under salt stress [102]. Subsequent studies further revealed that GmPHD6 can form a transcriptional complex with LHP1–1/2 to regulate the stress tolerance of *G. max* L. [103]. Specifically, GmPHD6 mainly recognizes and binds to the downstream *Cytochrome P450*-*71A22*/*82C4*/*75B1* (*CYP*-*71A22*/*82C4*/*75B1*) and *ABA Stress Ripening* (*ASR*) promoters. Moreover, the LHP1 protein is responsible for activating *CYP71A22*/*82C4*/*75B1* and *ASR* expression. *CYP71A22*/*82C4*/*75B1* as a member of a subfamily within the cytochrome P450 superfamily, overexpression of each gene can significantly increase the salt tolerance of *G. max L*. [103]. Another study revealed that the genome of *O. sativa* L. contains three genes encoding proteins of the TrxG family, namely *OsSDG723*, *OsSDG721* and *OsSDG705*. Among these genes, *OsSDG721* is induced to upregulate the expression of *High-Affinity K^+^ Transporter 1;5* (*OsHKT1;5*) under salt stress, thereby maintaining the K^+^/Na^+^ balance and enhancing the salt tolerance of *O. sativa* L. The *ossdg721* mutant is more sensitive to salt and alkaline stress, with a reduced survival rate and decreased plant height, grain size, grain weight, and leaf angle [22]. The tolerance of different *O. sativa* L. varieties to salt stress significantly differs. In salt-sensitive varieties (*IR64*), the H3K27me3 level upstream and in the 5′UTR of the *Basic leucine zipper 8* (*OsBZ8*) gene decreased, whereas H3K4me3 levels in the promoter region (−196 to −1) and the 200-bp region downstream of the transcription initiation site (TSS) significantly increased. In contrast, at the same sites in salt-tolerant varieties (Nonabokr), the H3K27me3 and H3K4me3 levels showed significantly opposite trends [104]. These results indicate that the differential expression of *OsBZ8* under salt stress is driven by locus-specific differences in H3K27me3 and H3K4me3 modification patterns. After salt stress treatment, H3K27me3 levels in *A. thaliana* significantly decreased, leading to the significant upregulation and maintenance of high expression levels of the *HKT1*, *Plasma Membrane Intrinsic Protein 2E* (*PIP2E*), and *Gretchen Hagen 3.1*/*3* (*GH3*.*1*/*3*) genes [105]. In *castor bean* seedlings, the expression of ABA-mediated *Radialis Adialis*-*Like SANT*/*MYB 1* (*RSM1*) plays a conserved role in the salt stress response, and its transcription is subject to bivalent modification of H3K4me3-H3K27me3 [106].

## Heat stress

Under high-temperature stress, HMTs and KDMs dynamically affect gene expression by regulating the methylation status of histones (Fig. 4). H3K36me3 regulates temperature-dependent gene-splicing events. The decreased levels of H3K36me3 in *A. thaliana sdg8* and *sdg26* mutants affect the splicing patterns of *Flowering Locus M* (*FLM*), *MAF3*, *SC35-Like SPLICING FACTOR 33* (*SCL33*), *U2 SnRNP Auxiliary Factor Large Subunit B* (*U2AF65B*), and *Pseudo Response Regulator 7* (*PRR7*), further promoting the expression of *FT* and accelerating flowering [107]. The expression levels of both *SDG25* and *ATX1* in *G. max* L. were significantly upregulated under high-temperature stress. The *sdg25* and *atx1* mutations led to a decrease in the level of H3K4me3, thereby reducing the expression of heat stress genes and increasing the sensitivity of the plants to high temperatures [108]. Another study revealed that high-temperature stress can lead to the accumulation of H3K4me3, thereby activating the transcription of *Heat Shock Protein 18*/*22*/*70* (*HSP18*/*22*/*70*) and *Ascorbate Peroxidase* (*APX2*) [109]. These added histone methylation markers can be removed by the JUMONJI (JMJ) protein family. In *A. thaliana*, the demethylases JMJ14 and JMJ15 were found to be recruited to the promoter region of *Cytosolic Isocitrate Dehydrogenases* (*clCDH*) under high-temperature stress, and their expression was further promoted by removing the H3K4me3 marker [110]. Another study revealed that the demethylase JMJ713, which is in the same family as JMJ14 and JMJ15, is also crucial in the high-temperature stress response of *O. sativa* L. *JMJ713* overexpression increased the heat tolerance of *O. sativa* L., whereas the *JMJ713*-silenced *O. sativa* strain presented increased sensitivity to heat. Another study revealed that *JMJ713* overexpression activates CAT and POD by reducing the excessive accumulation of ROS caused by heat stress [111]. Furthermore, both the JMJ30 and JMJ32 demethyltransferases can remove the H3K27me3 label in the promoter regions of *HSP22* and *HSP17*.*6C*, enabling them to be activated more quickly under subsequent high-temperature stress conditions to increase the tolerance of plants to high-temperature stress. These findings further reveal the important role of demethyltransferases in regulating the high-temperature tolerance of plants [112].

## Cold stress

Histone methylation forms a complex regulatory network in response to low-temperature stress, involving multiple transcription factors and epigenetic modification enzymes (Fig. 4). In potato (*Solanum tuberosum*), low-temperature stress leads to increased chromatin accessibility of gene activity in tubers, which is closely related to the bivalent modification of H3K4me3-H3K27me3 [113]. Another study revealed that low-temperature stress can lead to decreased levels of H3K27me3 in the promoter regions of *COR15A* and *AtGOLS3* in *A. thaliana*, thereby increasing the cold tolerance of the plants [114]. In chrysanthemum (*Dendranthema grandiflorum* var. *Jinba*), overexpression of *DgATX* significantly increased the overall H3K4me3 level and significantly improved the cold resistance of the plant. In contrast, the *dgatx* mutant shows a decrease in cold resistance. Further research revealed that DgMYB transcription factor can recruit DgATX to the promoter region of the downstream target gene *DgPOD* and promote its transcriptional, reducing the accumulation of reactive oxygen species (ROS) under low-temperature stress and thereby enhancing the adaptability of chrysanthemum to low-temperature stress [115]. After the strawberry (*Fragaria vesca*) seedlings were treated at 4°C for 3 h, the expressions of *ATX3b* and *SUVH4a* significantly increased, while *SUVH4*/*b*/*c*/*d* showed no response at all [116]. During the recovery period after low-temperature acclimation, the transient increase in the expression of the *Respiratory Burst Oxidase Homolog 5.1* (*CsRBOH5*.*1*) gene in cucumber (*Cucumis sativus* L.) was necessary to maintain the activity of NADPH oxidase and the content of extracellular H_2_O_2_, thereby enabling cucumber to acquire cold tolerance. This confirmed that CsRBOH5.1 was almost essential for the formation of a normal H3K4me3 deposition pattern in all encoding genes during the recovery process, and this necessity became more pronounced as the recovery time extended [117].

## Histone methylation–signal integration model

**Figure 5:**
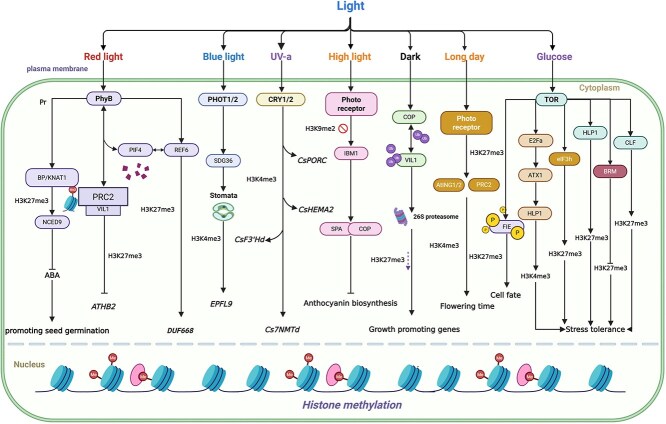
Model of plant histone methylation and signaling pathways. The red light receptor PhyB directly deposits H3K27me3 at the target gene locus by interacting with the VIL1–PRC2 complex, thereby inhibiting the expression of the growth-promoting gene ATHB2. TOR can activate downstream E2Fa and thereby increase the expression of ATX1, thereby maintaining the accumulation of H3K4me3 on heat stress response genes to establish and preserve heat stress memory. This model illustrates the interactions between histone methylation and light signals, TOR signals, and hormone pathways, as well as their cross-action in chromatin regulation mediated by methyltransferases, demethylases, and related chromatin complexes. How epigenetic mechanisms integrate multiple signals to coordinate plant development and environmental adaptation has been emphasized. “The publication license for BioRender has been obtained, and the work was created by BioRender.com.”

Activating markers, which are composed mainly of H3K4 and H3K36, and inhibitory markers, which are composed mainly of H3K27 and H3K9, are of vital importance in key life processes of plants such as dormancy and germination, flowering and senescence, and response to adverse conditions. However, these methylation markers are not accomplished independently but are regulated in a coordinated manner by multiple signaling networks. By reviewing the relevant research progress on histone methylation in recent years, we propose an integrated model of histone methylation with optical signals, TOR pathways, and hormone signaling pathways (Fig. 5), clarifying how histone methylation is interwoven with optical signals, TOR pathways, and hormone signals. Through the synergistic action of methylases, demethylases and related chromatin complexes, they jointly regulate the development process and environmental adaptability of plants.

Light signaling pathways are initiated by multiple types of photoreceptors. Phytochrome B (phyB), a photosensitive pigment, is among the main receptors for optical signal transduction and is directly functionally associated with the chromatin modification complex VIL1-PRC2 (Fig. 5). phyB can promote the deposition of H3K27me3 by interacting with the PRC2 component Vin3-like 1/VERNALIZATION 5 (VIL1/VRN5). Inhibition of *A. thaliana homeobox protein 2* (*ATHB2*) expression in a light-dependent manner [118]. However, the PhyB and VIL1 proteins themselves cannot bind to DNA. PhyB is likely to rely on transacting factors such as transcription factors to indirectly regulate the chromatin state. Recent studies further support the view that the activated Pfr form of phyB can preferentially interact physically with the demethylase REF6, increasing the accumulation of REF6 and its binding ability to target genes. It collaborates with REF6 and Phytochrome-Interacting Factor 4 (PIF) to regulate the expression of *DUF668*, thereby mediating the elongation of *A. thaliana* hypocotyls under light conditions and revealing a more direct molecular link between light signals and epigenetic regulation. It provides new insights into the complex interactions among photosensitive pigments, epigenetic factors, and transcription factors [119]. In addition to the PhyB, photosensitive proteins such as Phytotropins (PHOT1/PHOT2) and Cryptochromes (CRY1/CRY2) can affect chromatin structure and epigenetic modifications. Wang *et al.* [120] conducted genome-wide enrichment region analyses of six histone modifications in tea tree (*Camellia sinensis*) leaves treated with white light, blue light, and UV-A light through ChIP-seq technology. The key roles played by blue light and UV-A in histone H3K4 methylation mediated by histone CsSDG36 in the leaf development and secondary metabolism of tea tree (*C. sinensis*) plants were systematically revealed. This study further elucidated the role of photoreceptors in leaf development and secondary metabolism as well as their epigenetic mechanisms.

After activation, photoreceptors rapidly regulate the activities of a series of downstream core proteins, among which the COP1–SPA complex formed by *Constitutively Photomorphogenic 1* (*COP1*) and the *Suppressor of PHYA* (*SPA1*), *SPA3*, and *SPA4* genes is a key node for regulating anthocyanin biosynthesis in plants. Under high light, *IBM1* is upregulated, and the H3K9me2 inhibitory marker on the *SPA* gene is removed, thereby enhancing the inhibitory effect of the optical signaling pathway COP1–SPA on the anthocyanin pathway and achieving fine regulation of anthocyanin accumulation induced by high light [121]. VIL1/VRN5 is another substrate of COP1. The COP1-VIL1 module can specifically regulate the H3K27me3 levels of 665 growth-related genes and drive chromatin remodeling of related downstream genes, thereby regulating their expression and coordinating the plant’s response to changes in the external environment [122]. The influence of light signals on flowering regulation is carried out mainly through the H3K4me2/3-AtING1/2-PRC2 module. This regulatory mode enables the chromatin state to transition from activation to inhibition, thereby precisely controlling photoperiodic flowering. It provides an important potential target for the genetic improvement of flowering time in photoperiodic-sensitive plants [123]. In addition, direct functional connections exist between light signals, epigenetic modifications, and hormone regulation. Gu *et al.* [124] reported that the light signal stabilized Brevipedicellus/Knotty-like *A. thaliana* 1 (BP/KNAT1) through phyB, thereby increasing the H3K27me3 level of NCED6/9 and inhibiting ABA synthesis. Thus, the perception of external light is closely linked to epigenetic regulation and hormone metabolism, constituting the phyB-BP/KNAT1-H3K27me3-ABA regulatory mechanism that promotes light-induced seed germination.

TOR, as a core energy-sensing kinase for plant growth and metabolism, plays a central role not only in promoting growth and inhibiting stress responses, but also in directly regulating chromatin states (Fig. 5). Recent research has indicated that TOR also plays a key role in maintaining transcription-related epigenetic memory induced by heat stress in plants. Glucose, as a core signaling molecule for plants to perceive energy and nutritional status, can activate the TOR signaling pathway and promote the accumulation of H3K4me3 in the promoter of the heat stress response gene *Hikeshi*-*like protein 1* (*HLP1*), helping plants acquire heat tolerance [125]. The glucose–TOR–E2Fa–ATX1 signaling pathway constitutes the core molecular mechanism for regulating heat memory. Among them, TOR activates downstream *E2Fa* and thereby increases the expression of *ATX1*, thereby maintaining the accumulation of H3K4me3 on heat stress response genes to establish and maintain heat stress memory [126]. After activation by glucose, TOR phosphorylates FIE and promotes changes in its localization change from the cytoplasm to the nucleus, increasing the H3K27me3 level mediated by PRC2 and thereby silencing key transcription factors that regulate cell fate determination, developmental transitions, and organ patterns in *A. thaliana* to regulate various developmental transitions in plants [77]. Furthermore, under conditions of adequate nutrition and high TOR activity, TOR promotes the epigenetic inhibitory functions of the key components of PRC2, *CLF* and *LHP1*; increases the modification level of H3K27me3; and simultaneously inhibits the activity of the chromatin remodeling factor *Brahma* (*BRM*), silencing stress response genes and prioritizing their support for plant growth. When plants encounter adverse conditions, TOR activity decreases, the function of the PRC2 complex is inhibited, H3K27me3 modification is reduced, stress response genes are activated, and a defense response is initiated [127]. Studies have shown that the *CLF* gene in *A. thaliana* is regulated by TOR through EIF3h-dependent translational reactivation. Among them, the 5′utr uORF of *CLF* is the key sensing element of this regulation and the dynamic regulation core of the TOR–PRC2 pathway [128]. This study elucidates the molecular pathway between TOR–uORF–eIF3h and PRC2-mediated H3K27me3, revealing that plants achieve rapid transcriptional reprogramming in response to environmental signals through dynamic regulation of the plasticity of H3K27me3, providing a key breakthrough in the integration mechanism of metabolic state and epigenetic regulation.

## Conclusions and prospects

With the integrated application of whole-genome sequencing and proteomics analysis techniques, breakthroughs have been made in research on the working mechanisms of epigenetic regulation in plant development and environmental adaptability.

Histone methylation, as the core mechanism of the epigenetic regulatory network, plays important roles in key life activities, such as plant dormancy and germination, flowering and senescence, and the stress response. Histone methylation enzymes can regulate not only springtime and photoperiod-induced plant flowering through crosstalk with flowering- influencing factors (*CO*, *FT*, *SOC1*, etc.) but also are an ideal epigenetic resource for improving plant architecture [129,130]. Recent studies have revealed that histone methylation not only affects development and metabolic processes but also strongly participates in the immune regulation of plants against biological stress. The histone lysine methyltransferases SDG8 and SDG25 synergically regulate the immune response of plants to pathogenic bacteria at multiple levels such as transcription, metabolism, and structure by regulating the methylation status of H3K4 and H3K36. They are key epigenetic regulatory factors for innate immunity and systemically acquired resistance in plants [131]. However, due to problems such as functional redundancy and/or lack of specificity of histone methylation modification enzymes, it is difficult to achieve precise editing of specific modifications, thereby limiting their direct application in molecular breeding [132]. Oberkofle *et al.* [133]constructed an inducible, site-specific epigenome editing system. This system can specifically target JMJ18 to the promoter region of the target gene *APX2*. Through ChIP experiments, it was confirmed that the dCas9-JMJ18 protein could be successfully recruited to a specific location of the gene after heat stress, resulting in a significant decrease in the H3K4me3 methylation level at the *APX2* site induced by heat stress. This achieved ‘knocking down’ of a certain histone modification at a specific time and specific gene locus, overcoming the limitations of traditional genetic methods due to functional redundancy and pleiotropy, and laying the foundation for a deeper understanding of the core role of epigenetic regulation in plant life activities. In addition, the chromatin state is regulated by multiple upstream signals such as TOR, light signals, and hormones, thereby precisely controlling key physiological processes such as the release of plant dormancy, the initiation of flowering, and adaptation to adverse conditions. Therefore, future research urgently needs to start from the perspective of chromatin and metabolic signal integration and systematically analyse the dynamic mechanism of epigenetic regulation under multisignal intersection in order to deepen the understanding of the plant environmental adaptability regulatory network and provide a theoretical basis for the precise improvement of horticultural plants.

Although the correlation between histone methylation modification and transcriptional activity has been established, the proteins and complexes that interact with histone methylation enzymes and how they are specifically recruited to specific sites remain unclear. Recent research has indicated that, unlike the traditional strategy of knocking out histone methylation modification enzymes, the targeted histone amino acid replacement technology can directly reveal the biological function of the modification at a specific site by precisely mutating it, thereby achieving a comprehensive analysis of epigenetic markers. This technology has been successfully applied in *A. thaliana* research, for instance, by mutating the lysine at position 36 of histone H3 to methionine (H3K36M), or by replacing the specific lysine K27 on histone variant H3.3 with alanine K27A. By replacing the specific lysine K4 on the histone variant H3.3 with alanine K4A, arginine K4R, and glutamine K4Q, respectively, the system revealed the functional specificity and regulatory differences of these sites under different chemical states [134,135,136]. Looking ahead, the combination of targeted histone amino acid substitution technology with novel epigenomic tools will help to systematically analyse the comprehensive impact of specific amino acid substitutions on histone modifications, chromatin state, and gene expression across the entire genome, thereby establishing a causal relationship between histone modifications and biological functions in multiple dimensions. Future research should focus on applying these techniques in more complex physiological contexts or under environmental stress to explore the profound impact of key histone residue replacement on plant phenotypes and chromatin remodeling under specific environmental conditions.

With the continuous enrichment of research on the regulation of plant gene expression by single histone methylation modifications, bivalent histone modifications of histone methylation, i.e. the coexistence of activating (H3K4me3) and inhibitory (H3K27me3) methylation markers on lysine residues of the same histone, has received increasing attention. This bivalent histone modification is different from the single histone methylation that maintains a gene in an activated or inhibited state. Instead, it is more inclined to a built-up state. Once a plant’s own developmental state changes or is stimulated by external environmental signals, bivalent histone modifications can quickly respond to the signals and shift to an activated or inhibited state. It has been reported that the TOR is a central hub connecting various external environmental stimuli and gene expression. In *A. thaliana*, highly active TOR kinase maintains the function of CLF/LHP1, promoting the deposition of the inhibitory markers H3K27me3 on genes such as *JAZ1* and *WRKY70*, thereby maintaining their transcriptional silencing state and enabling them to exhibit a phenotype that promotes vegetative growth. Conversely, when TOR activity decreases, the level of H3K27me3 decreases significantly. These genes, which are in a ‘standby’ state because of the preexisting activation marker H3K4me3, are rapidly released, and their expression is sharply upregulated, thereby quickly initiating the defense program and enhanced the resistance of *A. thaliana* to pathogens such as gray mold fungus [137]. This bivalent modification pattern independently regulates the transcription of genes, rather than compete with each other [138]. Further in-depth mechanism studies have shown that this bivalent histone modification can be mediated by a single protein, such as the plant-specific histone reader enzyme SHL, which can recognize H3K27me3 and H3K4me3 through the BAH and PHD domain, respectively, and is different from other modules in terms of recognizing active and inhibitory histone markers [139]. Zeng *et al.* [113] performed whole-genome analysis and identified many novel low-temperature-induced H3K4me3–H3K27me3 bivalent chromatin regions in potato, providing new insights into the biological functions of bivalent methylations in plants. Another study revealed that the bivalent modification of H3K4me3–H3K27me3 induced by plant cold stress is related to the participation of *ATX1* and *CLF* and is completely different from the inhibitory mechanism in mammals [140]. In addition to the bivalent H3K4me3–H3K27me3 modification mediated by ATX1 and CLF, the H3K4me3–H3K9me3/2 mediated by Spindlin1 also regulates gene expression. During genomic reprogramming, H3K4 methyltransferase can deposit H3K4me3 in heterochromatin regions that were originally rich in H3K9me3/2. H3K4me3–H3K9me3/2 can then specifically recruit Spindlin1, thereby maintaining downstream genes in a ‘suspended’ expression state to respond rapidly to external cell differentiation or metabolic signals [141]. The fruit of strawberry (*F. vesca*) has ‘bivalent modification’ functionally similar to those reported in animals and plants, enabling it to respond rapidly to ripening signals (ABA) and achieve coordinated activation of genes through chromatin state transitions, thereby precisely regulating the fruit ripening process [142]. However, when this concept is extended to polyploid horticultural species with more complex genomes, we are confronted with unique challenges and opportunities. In polyploid species, there are multiple copies of homologous genes, which may have functional redundancy, subfunctionalization, or even functional differentiation. This redundancy makes functional verification through means such as gene editing extremely difficult, and multiple copies need to be knocked out simultaneously to observe phenotypic changes. To overcome these obstacles and unlock the potential of epigenetic regulation in crop improvement, we urgently need to conduct integrated studies at key developmental stages of multiple polyploid horticultural crops, map species-specific epigenomic maps of histone modifications, DNA methylation, and chromatin accessibility, and provide new operational precision breeding strategies for complex genome crops. At present, there are still few studies on the bivalent modification mechanism of histone methylation. The existing work focuses mainly on elaborating the correlation between the bivalent modification state and the gene expression level. Future research needs to focus deeply on the divalent promoter region and systematically analyse the specific molecular mechanisms through which bivalent modification dynamically regulates plant growth and development as well as stress response processes.

In addition, the current research on the synergistic or antagonistic mechanisms between histone methylation modifications and other types of epigenetic modifications (such as histone acetylation, ubiquitination, SuMOylation, etc.) is not yet in-depth. The existing achievements are mostly focused on revealing the molecular cross-slicing mechanisms between histone methylation and DNA methylation. Li *et al.* [143] reported that the demethylase SlJMJ6 in tomato removes H3K27me2/3 and significantly upregulates the expression levels of *Ripening Inhibitor* (*RIN*), *1*-*aminocyclopropane 1*-*carboxylate synthase*-*4* (*ACS4*), *1*-*aminocyclopropane*-*1*-*carboxylate oxidase 1* (*ACO1*), *pectate lyase* (*PL*), *beta*-*galactosidase 4* (*TBG4*), and *DNA demethylase* (*DML2*), thereby promoting fruit ripening. These findings clarified that histone methylation and DNA methylation involve molecular crosstalk at the transcriptional level. Notably, subsequent studies have further revealed the reverse regulatory mechanism of JMJ7, a member of the JMJ family, in the fruit ripening of tomatoes. SlJMJ7 specifically removes the epigenetic marker H3K4me3 and directly targets and inhibits *SIDML2* expression. The level of H3K4me3 in the promoter region of *SIDML2* shows a dynamic balance with the activity of SlJMJ7 [144]. H3K4me3- and SIDML2-mediated DNA methylation results in the formation of an epigenetic cascade network that synergistically regulates gene expression. Further research revealed that SUVH4 specifically recognizes DNA methylation markers, catalyses the dimethylation of the histone H3K9 site, and then recruits the DNA methyltransferase CMT3 to the CHG site to maintain methylation. Ultimately, a positive feedback regulatory network between DNA methylation and H3K9me2 is formed [145]. The deletion of DNA methylation in *A. thaliana* mutants leads to the complete loss of H3K9me2 and the extensive redistribution of H3K27 [141]. Compared with the model plant *A. thaliana*, the research foundation of horticultural crops in the field of histone methylation modification is still relatively weak. Therefore, systematically drawing on the mature research achievements of other species in this field will effectively expand the depth and breadth of histone methylation research in horticultural plants, and provide a solid theoretical support for formulating new, targeted crop design breeding strategies based on epigenetic regulation in the future.
